# Elucidating the significant roles of root exudates in organic pollutant biotransformation within the rhizosphere

**DOI:** 10.1038/s41598-024-53027-x

**Published:** 2024-01-29

**Authors:** Michael O. Eze, Chinedu F. Amuji

**Affiliations:** 1 Department of Chemistry, and Metabolomics and Environmental Toxicology Laboratory, Missouri University of Science and Technology, Rolla, MO 65409 USA; 2 Centre for Research in Energy and Environment, Missouri University of Science and Technology, Rolla, MO 65409 USA; 3 Department of Crop Science, University of Nigeria, Nsukka, Enugu State Nigeria

**Keywords:** Pollution remediation, Bioanalytical chemistry, Environmental sciences

## Abstract

Biotransformation of organic pollutants is crucial for the dissipation of environmental pollutants. While the roles of microorganisms have been extensively studied, the significant contribution of various root exudates are still not very well understood. Through plant growth experiment, coupled with gas and liquid chromatography-mass spectrometry methods, this study examined the effect of the presence of *M. sativa* on microbial-associated biochemical transformation of petroleum hydrocarbons. The results of this study revealed that the concentration of exudates within the soil matrix is a function of proximity to root surfaces. Similarly, biodegradation was found to correlate with distance from roots, ranging from ≥ 90% within the rhizosphere to < 50% in bulk soil and unplanted control soil. Most importantly, for the first time in a study of an entire petroleum distillate, this study revealed a statistically significant negative correlation between root exudate concentration and residual total petroleum hydrocarbons. While not all the compounds that may influence biodegradation are derived from roots, the results of this study show that the presence of plant can significantly influence biodegradation of hydrocarbon pollutants through such root exudation as organic acids, amino acids, soluble sugars and terpenoids. Therefore, root exudates, including secondary metabolites, offer great prospects for biotechnological applications in the remediation of organic pollutants, including recalcitrant ones.

## Introduction

Anthropogenic pollution of the environment continues to be on the increase, especially with population growth and industrialization. Even beneficial activities such as pest control in agriculture and fossil fuel exploitation always leave behind unwanted and oftentimes toxic effects. Phytoremediation, the use of plants and associated microorganisms to remove contaminants, is regarded as a low-cost and eco-friendly alternative to traditional site clean-up methods and has thus gained increased attention in recent decades. In phytoremediation of organic and inorganic pollutants, plants can contribute to remediation through processes such as phytovolatilization (transformation of contaminants into volatile compounds and their release to the atmosphere through evapotranspiration), phytoextraction (accumulation of contaminants in harvestable parts), phytostabilization (sequestration or immobilization of contaminants in root cells), and rhizodegradation (breakdown of contaminants into simpler and often non-toxic compounds)^1^. While plants are able to produce contaminant-degrading enzymes, this process is generally a minor contributor to organic pollutant dissipation in the environment^2^. Rhizoremediation relies heavily on biodegradation of contaminants through the activities of rhizosphere microbes. These activities mainly take place within the rhizosphere.

As described by Lorenz Hiltner in 1904, the rhizosphere refers to the area surrounding plant roots, where processes mediated by microorganisms, and central to plant health, take place^3^. It represents a highly dynamic environment involving complex plant–microbe interactions. The rhizosphere effect describes the phenomenon where, in comparison to bulk soil, the biomass and activity of microorganisms is enhanced by an order of magnitude through the exudation of compounds by plant roots^4^. The biological and physico-chemical interactions between roots and rhizosphere microbes are among the most complex mechanisms affecting plants. These processes exert tremendous impact on plant and microbial health. In recent decades, the rhizosphere and associated biochemical process has been a subject of intense investigation owing to the effect of the rhizosphere on such processes as nutrient availability, microbial community dynamics, carbon sequestration, contaminant biodegradation and bioremediation^5–8^. Under a variety of conditions, it has been demonstrated that vegetation enhances microbial degradation of environmental pollutants^9,10^.

A review of literature reveals that root exudates orchestrate the complex interactions taking place in the rhizosphere and are thus regarded as the driving force for rhizoremediation. This is because rhizosphere microorganisms in polluted sites generally live under conditions of nutrient starvation and are constantly looking for nutrients^1^. Therefore, root exudates such as organic acids, amino acids, and sugars provide microbes with nutrients needed for biodegradation. On the other hand, some rhizosphere microbes can contribute to the growth of host plants through processes such as nitrogen fixation, phosphate and potassium solubilization, and phytohormone synthesis^11,12^. This synergistic interaction not only multiplies the rhizosphere effect by several orders of magnitude but also enhances biodegradation of environmental pollutants. For example, in a recent study involving the plant growth promoting bacteria *Paraburkholderia tropica* WTPI1, it was found that bacteria-plant synergistic interactions resulted in a 96% rhizodegradation of petroleum hydrocarbons as opposed to 49% under natural attenuation^11^. Corgié, et al.^13^ observed that phenanthrene biodegradation was a function of proximity to roots, and that the gradient of degradation correlated with that of microbial density.

While there is a growing interest in unraveling the rhizosphere microbiome, understanding the complexity and drivers of rhizospheric biochemical interactions remains a challenge^14^. In view of the important role of the rhizosphere on plant health and its potential to enhance biodegradation of environmental organic pollutants, this study was aimed at examining the effect of the rhizosphere and root exudate concentration on the dissipation of diesel fuel hydrocarbons along a soil matrix from *Medicago sativa* L. roots to the bulk soil. Although a few studies have examined the degradation of individual compounds such as phenanthrene in relation to root proximity^2,13^, to the best of our knowledge, this is the first study of the relationship between root exudates and residual total petroleum hydrocarbons of an entire petroleum distillate (diesel fuel). The results of this study revealed that the degradation of diesel fuel hydrocarbons decreased with increased distance from the root, with greater than 90% biodegradation observed in soils directly attached to the roots. Most importantly, for the first time, this study revealed a statistically significant negative correlation between root exudate concentration and residual total petroleum hydrocarbons, indicating that biodegradation correlates positively with the concentration of exudates, including organic acids, amino acids, soluble sugars and terpenoids.

## Materials and methods

### Soil preparation

The soil used for this experiment was “Primaster turf”^10^ obtained from REWE Group, Goettingen, Germany. Primaster turf is a mixture of screened sand, soil, and composted organics. It is blended with a nitrogen-phosphorus-potassium fertilizer to promote root growth throughout the year. The soil texture was sand (88.6% sand, 6.1% silt and 5.3% clay), with 12.5% organic matter content measured by loss on ignition. The soil had a total nitrogen content of 0.15% and a pH of 7.1. The soil was sieved using a 2-mm sieve to remove large particles. Diesel fuel (C_10_–C_25_), specifically petrodiesel, obtained from Shell service station, Goettingen, Germany was added to the soil and homogenized following the methods of Eze, et al.^15^ with minor modifications. The modifications involved changing the duration of the different stages involved in the homogenization process. In brief, the soil was manually homogenized for one hour. This was followed by automatic homogenization using a portable soil mixing machine (Güde Model GRW 1400) for 30 min. The soil was allowed to age for one week. Gas chromatography–mass spectrometry analysis revealed that the resulting total petroleum hydrocarbons concentration in the diesel fuel-contaminated soil was 5.30 g/kg.

### Assessment of seed viability

The triphenyltetrazolium chloride (TTC) test has been developed to provide a rapid estimate of seed viability^16–18^. TTC is a clear, water-soluble compound (a salt) which is reduced by respiring tissues to yield triphenylformazan (TPF), a water-insoluble red pigment. Thirty seeds of *M. sativa* L. were subjected to the TTC test as follows. Each batch of seeds was placed in a beaker containing 50 mL of 1% TTC, prepared by dissolving 1 g of TTC in 100 mL distilled water. The beakers were covered and placed in an incubator at 30 °C for 1 h. Following incubation, the liquid was decanted, and the seeds were rinsed with distilled water until the water was clear. The seeds were blotted with dry towel and the colour was observed. The seeds were classified into two categories according to their colour development as described in Eze, et al.^15^, namely: “red/pink” and “no colour”, corresponding to “viable” and “not viable”, respectively. The batch with almost 100% viability was used for the plant growth experiment. The current study, including the collection of plant materials (seeds of *M. sativa* L.) followed Macquarie University and Georg-August University of Goettingen guidelines and legislation.

### Plant growth experiment

Based on the results of TTC test, rhizosphere study was conducted using a series of pot experiments in a greenhouse following the methods of Eze et al.^10^. Pots containing 300 g of diesel fuel contaminated soils were planted with two *M. sativa* seeds (Supplementary Fig. 1). Following germination, one seedling was removed from each pot, thereby leaving a seedling per contaminated pot. This was done to make it easier for rhizosphere soil sampling later in the experiment. An unplanted contaminated pot served as the control (no rhizosphere effect). The whole experiment was performed in triplicates. Since the goal of the study was to assess the effect of rhizosphere effect on the dissipation of hydrocarbons through biodegradation, the soil used for the entire experiment was the diesel fuel-contaminated soil (5.30 g/kg) as described above. Pots were watered with 120 mL sterile water every three days for the first two weeks. After that, the planted pots were watered with 120 mL sterile water every two days to compensate for the water needs of *M. sativa* plants, without allowing leaching out of hydrocarbons. To assess growth rate of *M. sativa* in contaminated soils, shoot heights, from shoot tips to the base of stem^15,19^, were measured every two weeks. Prior to rhizosphere soil sampling, pots were left without watering for three days, allowing soils to adhere strongly to the roots. At harvest, the pots were ripped gently to prevent below-ground soil from disintegrating. Soils were taken from various parts of the pot as follows: (1) soil attached to the roots; (2) soil at 1 cm from the roots; (3) soil at 3 cm from the roots, also known as the bulk soil. To enable accurate measurement of soil distances from the roots, one side of the adhering soil was gently scrapped thereby exposing the roots and associated rhizosphere and bulk soils. Soil distances from *M. sativa* roots were then determined using a line gauge (ruler). Bulk soils at 3 cm from the roots were collected first. Thereafter, rhizosphere soil at 1 cm from the roots were collected by gentle scrapping. Finally, soil attached directly to the roots was shaken off and collected. Each plant was then washed under tap water, oven-dried at 70 °C until constant weight was achieved. The dry biomass was recorded.

### Analysis of root exudates

Amino acids, organic acids and soluble sugars are important exudates that can impact the growth and metabolic activities of rhizosphere microbes. In view of the destructive nature of sample collection for root exudate analysis, the analysis of root exudates was performed at one timepoint (day 90). In addition, since rhizosphere sample collection often impacts the rhizosphere environment and potentially root exudation, choosing one timepoint helped eliminate such external impact and associated bias. To extract these exudates, soil samples (5 g) were added to a 250 mL flask containing 100 mL of distilled water containing the bactericide oxytetracycline hydrochloride (15 μg/mL) to prevent microbial degradation of exudates. The flasks were covered and shaken on a mechanical shaker at 110 rpm for 8 h at 30 °C (INFORS HT shaker, model CH-4103, Infors AG, Bottmingen, Switzerland). The extract was centrifuged at 4000×*g* for 20 min and filtered through a Nalgene 0.45-µm nylon membrane filter. The extract was then stored at −20 °C until later analysis. This approach is a modification of the methods of Xu, et al.^20^ and Gao, et al.^2^.

The root exudates were analyzed using an Agilent 1290 series HPLC coupled with an Agilent 6530 quadrupole time-of-flight (Q-TOF) mass spectrometer (Agilent Technologies, Santa Clara, CA, USA). An Agilent InfinityLab Poroshell 120 hydrophilic interaction chromatography (HILIC-Z) column (2.1 mm × 100 mm, ID: 2.7 µm) held at 25 °C was used to separate compounds. Mobile phases consisted of water (A) and 90% acetonitrile in water (B). Each mobile phase contained 10 mM ammonium acetate in water at pH 9 and a 5 µm deactivator additive (Agilent P/N 5191-4506). Compound separation occurred under a solvent gradient (0–2 min 95%B; 2–12 min 95%–50%B; 12–13 min 50%–95%B; 13–21 min 95%B) at a flow rate of 0.25 mL/min during a total run of 21 min per injection. Autosampler temperature was maintained at 10 °C with injection volume of 1 µL. An Agilent Jet Stream nebulizer was used as electrospray ionization (ESI) source. Samples were run in negative mode with mass range of 50–1000 *m/z* at gas temperature of 250 °C with capillary (ionization) and fragmentor voltages of 3000 V and 100 V respectively. Nebulizer gas pressure, temperature and drying gas flow rate were set at 40 psi, 300 °C and 12 L/min. Acquired data were treated with Agilent Mass Hunter B.06.00 software, and compounds were quantified using standard calibration curves.

### Organic geochemical analysis of biodegradation

At the end of the experiment, geochemical analysis followed the methods described in Eze, et al.^10^. Soils were freeze-dried and 1 g of the dried soil was further homogenized with a small amount of sodium sulfate (Na_2_SO_4_) and transferred into a Teflon microwave digestion vessel for hydrocarbon analysis. The samples were solvent extracted twice with 2.5 mL portions of freshly distilled *n*-hexane in a microwave device (Mars Xpress, CEM; 1600W, 100 °C, 20 min). For reference, 2.5 µL diesel fuel (density = 0.82 g/mL) was dissolved in 5 mL *n*-hexane instead of 1 g soil sample. The extracts for each sample were combined into a 7 mL vial and topped to 5 mL with *n*-hexane. A 1 mL aliquot (20%) of each extract was pipetted into a 2 mL autosampler vial, and 20 µL *n*-icosane D42 (200 mg/L) was added as an internal quantification standard.

Gas chromatography-mass spectrometry (GC–MS) analyses of the samples were performed using a Thermo Scientific Trace 1300 Series GC coupled to a Thermo Scientific Quantum XLS Ultra MS. The GC capillary column used was a Phenomenex Zebron ZB-5MS (30 m, 0.1 µm film thickness, inner diameter 0.25 mm). Compounds were transferred splitless to the GC column at an injector temperature of 300 °C. Helium was used as the carrier gas at a flow rate of 1.5 mL/min. The GC temperature program was as follows: 80 °C (hold 1 min), 80 °C to 310 °C at 5 °C/min (hold 20 min). Electron ionization mass spectra were recorded at 70 eV electron energy in full scan mode (mass range *m/z* 50–600, scan time 0.42 s). Peak areas were integrated using Thermo Xcalibur software version 2.2 (Thermo Fisher Scientific Inc., USA).

### Biodegradation parameters

To assess the nature and extent of biodegradation in the different treatments, the ratios of more refractory isoprenoid hydrocarbons pristane (2,6,10,14-tetramethylpentadecane, Pr) and phytane (2,6,10,14-tetramethylhexadecane, Ph) versus *n*-heptadecane (*n*C_17_) and *n*-octadecane (*n*C_18_) respectively were obtained. As an additional parameter, the ratio of the unresolved complex mixture (UCM, also known as the “hump”, an indicator of biodegradation^21^) versus total petroleum hydrocarbons (TPH) was determined.

### Statistical analysis

All statistical analysis were performed using R^22^. Relative growth rate of *M. sativa* plants were determined using the methods of Eze, et al.^15^. This method involved the measurement of growth rate in terms of shoot height attained with time. This approach eliminates the biases associated with the destructive harvesting method^23^. One-way analysis of variance (ANOVA) was used to compare the mean residual total petroleum hydrocarbons at different sampling distances from root surfaces with that of the control soil, followed by Tukey’s all-pairwise comparisons. In all cases, the normality of variances was tested by the Shapiro–Wilk’s method^24^, while homogeneity of variances was tested using Levene’s test^25^. Differences were considered significant at *p* < 0.05. The *p* values were adjusted using the Holm method to control family-wise error rate^26,27^.

## Results

### Seed viability and plant growth

The result of triphenyltetrazolium chloride (TTC) test revealed that more than 80% of the seeds were viable, evidenced by their red/pink colour at the end of the TTC test. The assessment of the growth of *M. sativa* in diesel fuel-contaminated soil showed that during the 90-day experimental period, *M. sativa* grew to a height of 91 ± 3 cm and a biomass of 5.80 ± 1.52 g (Supplementary Fig. 2).

### Concentration gradient of root exudates in the rhizosphere

**Figure 1:**
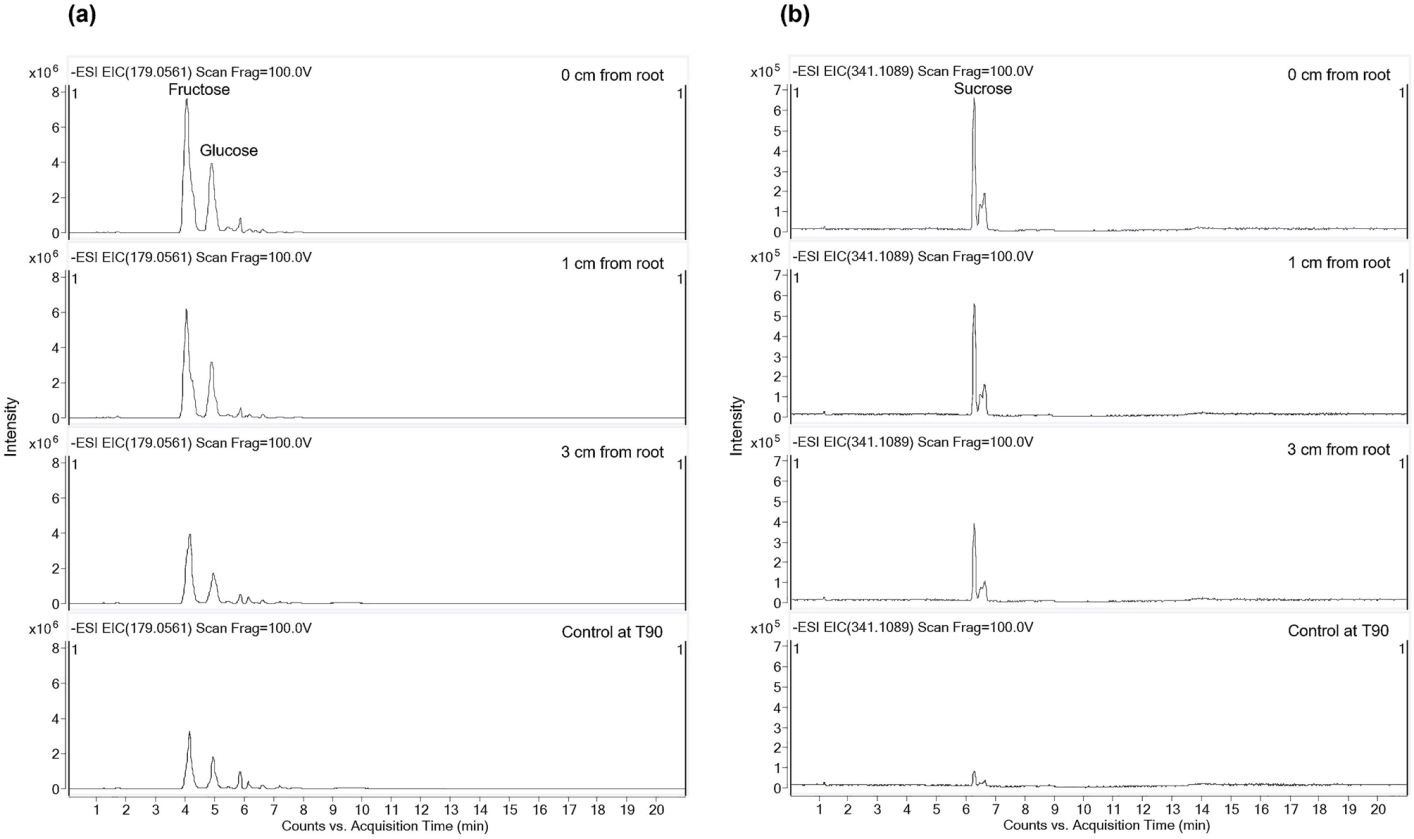
Extracted LC–MS ion mass chromatogram showing concentration gradient of (**a**) fructose and glucose, and (**b**) sucrose with increasing distance from plant root surfaces.

LC–MS analysis of root exudates revealed that the concentrations of amino acids (lysine, threonine and tryptophan), organic acids (acetic, oxalic and malic acids) and soluble sugars (fructose, glucose and sucrose) decreased with increased distance from root surfaces (Table 1). For example, the concentration of fructose and glucose, which serve as nutrients for most rhizosphere microbes, were 4.20 ± 0.13 mg/kg and 2.93 ± 0.12 mg/kg respectively in soils attached to *M. sativa* roots (0 cm from root). In planted soils, these values steadily declined along a gradient of distance away from root surfaces (Fig. 1) reaching the values of 1.99 ± 0.13 mg/kg for fructose and 1.65 ± 0.05 mg/kg for glucose at 3 cm from root surfaces. The lowest concentrations of all root exudate compounds at the end of the experiment were observed in the unplanted control soil (control at T90). The negative correlation observed between the concentration of root exudates and distance from root surfaces is an indication of rhizosphere effect in *M. sativa* planted soils (Supplementary Fig. 3).

**Table 1:** Concentration gradient of selected root exudates (mg/kg) at different distances from *M. sativa* root surfaces.

Root exudate	Distance from root surfaces			Control at T90
	0 cm	1 cm	3 cm	
Lysine	152.47 ± 5.15	136.66 ± 5.24	80.33 ± 4.26	72.46 ± 4.49
Threonine	202.62 ± 4.09	190.00 ± 4.62	130.67 ± 4.33	103.17 ± 4.38
Tryptophan	161.17 ± 5.23	152.57 ± 4.19	100.20 ± 4.02	96.64 ± 5.00
Acetic acid	140.67 ± 4.06	122.90 ± 5.31	79.07 ± 4.35	45.23 ± 4.05
Malic acid	181.73 ± 3.14	176.40 ± 4.16	90.67 ± 5.04	66.90 ± 4.27
Oxalic acid	207.13 ± 3.89	179.00 ± 4.58	104.78 ± 3.90	95.03 ± 3.60
Fructose	4.20 ± 0.13	4.12 ± 0.25	1.99 ± 0.13	1.41 ± 0.09
Glucose	2.93 ± 0.12	2.59 ± 0.15	1.65 ± 0.05	0.95 ± 0.06
Sucrose	1.71 ± 0.02	1.63 ± 0.05	0.85 ± 0.03	0.20 ± 0.02

It was also found that, with the exception of malic acids, the ability of amino acids and organic acids to migrate from root surfaces through soil matrix decreased (high slope values) with decreasing solubility of compounds belonging the same class (Supplementary Table 1). For example, the slopes of the plots of concentration versus distance for lysine, threonine and tryptophan were 0.76, 0.88 and 0.98 respectively. This corresponded to the decreasing solubilities of these compounds in water at 25 °C, namely 1000 g/L for lysine^28^, 97 g/L for threonine^29^ and 11.4 g/L for tryptophan^30^.

### Residual total petroleum hydrocarbons

**Figure 2:**
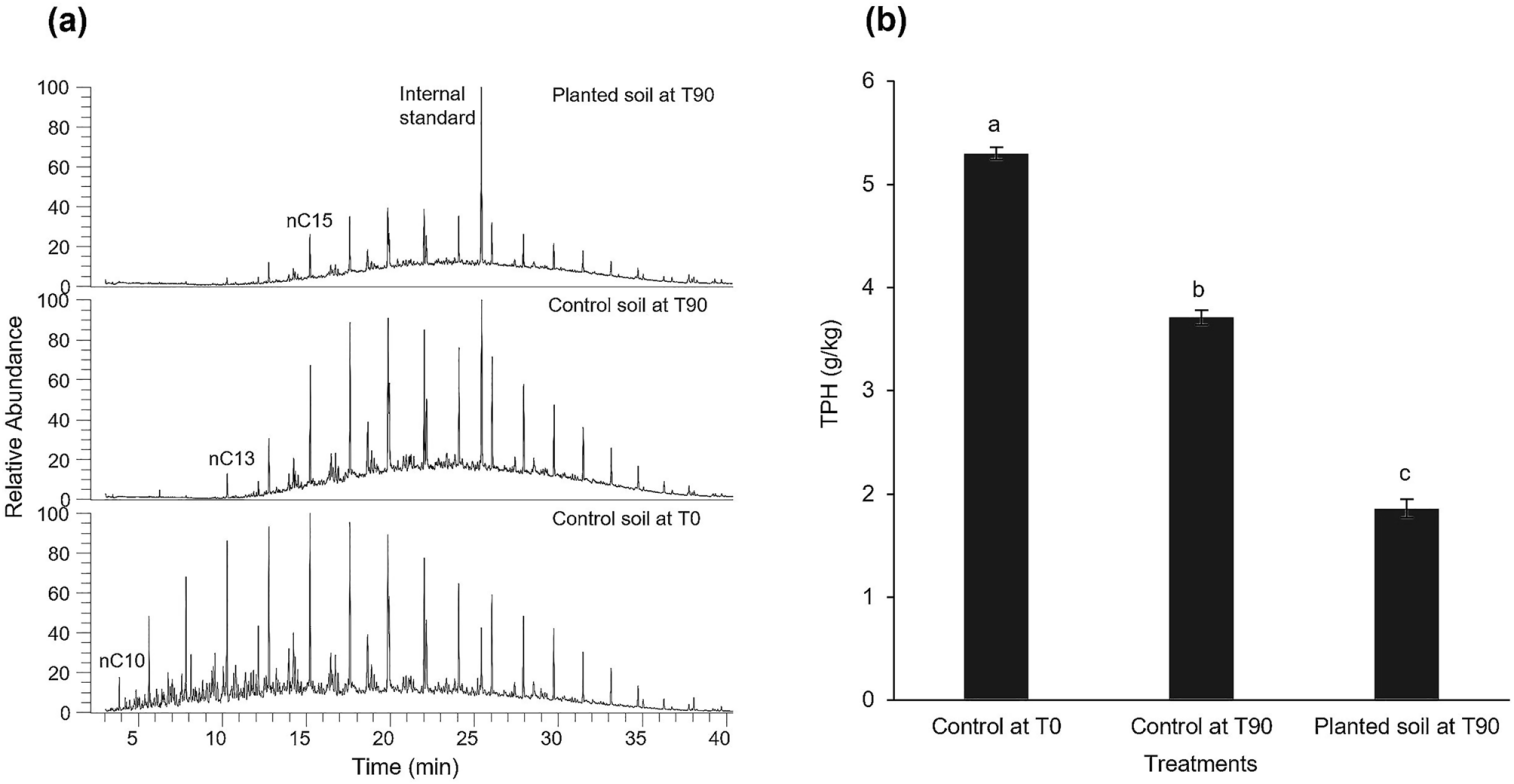
Phytoremediation of diesel fuel-contaminated soils as shown by (**a**) GC–MS total ion chromatograms showing the total hydrocarbons in the contaminated soil at the beginning of the experiment (T0) and the residual hydrocarbons after 90 days (T90) for control (unplanted) and planted soils. The chromatograms are normalized to sample weight and can be directly compared with reference to the internal standard. (**b**) Bar chart showing the mean values (± SE) of the initial and residual total petroleum hydrocarbons under different treatments (TPH, g/kg). Different letters indicate significant differences (*p* < 0.05).

Organic geochemical analysis of residual total petroleum hydrocarbons (TPH) revealed that phytoremediation with *M. sativa* significantly enhanced the dissipation of diesel fuel hydrocarbons in contaminated soils (Fig. 2). The mean residual TPH were 3.71 ± 0.07 g/kg for control soil and 1.86 ± 0.09 g/kg for planted soil (Fig. 2b). Statistical analysis of variance revealed significant differences between treatments (Fig. 2b).

### Phytoremediation as a function of contaminant proximity to root surfaces

**Figure 3:**
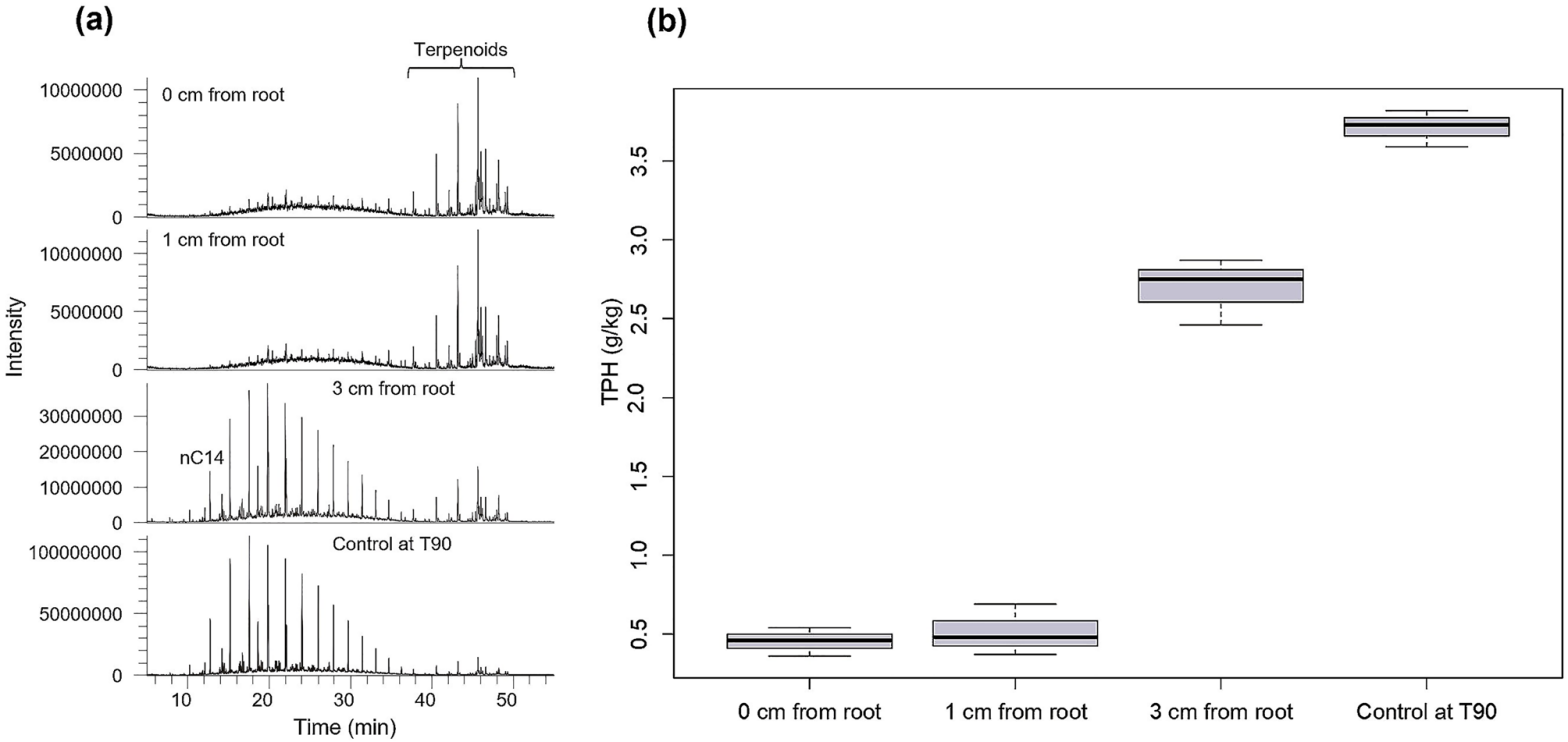
(**a**) Partial *m/z* 57 mass chromatograms showing differential degradation of petroleum hydrocarbons along a gradient from *M. sativa* root surface. The chromatograms are normalized to sample weight and can be directly compared with reference to intensity values. (**b**) Boxplot showing mean values (± SE) of residual total petroleum hydrocarbons at varying distances from plant roots in comparison with that of the unplanted soil (control at T90) at the end of the experiment.

The analysis of residual hydrocarbons at different distances from root surfaces revealed that biodegradation decreases with increasing distance from *M. sativa* roots (Fig. 3a), signifying that phytoremediation was a function of contaminant proximity to plant roots. The lowest mean level of residual TPH (0.45 ± 0.05 g/kg) was observed in soils directly attached to the roots (0 cm from the root), while the highest mean level (2.69 ± 0.12 g/kg) in planted soils occurred in the bulk soil (represented by soil at 3 cm from the root). Soils at 1 cm from *M. sativa* root surfaces gave mean residual TPH level (0.51 ± 0.09 g/kg) similar to that of soils directly attached to the roots (Fig. 3b). These concentrations represented 91%, 90%, and 49% biodegradation of diesel fuel hydrocarbons in contaminated soils at 0 cm, 1 cm, and 3 cm from root surfaces respectively.

One-way analysis of variance showed that the mean residual TPH in the planted soils at different distances (0, 1 and 3 cm) were significantly different (*p* = 4.74e-09, 5.50e-09 and 3.60e-05 respectively) from the residual TPH of the control soil (control at T90) at the end of the experiment, indicating that rhizosphere effect was responsible for the varied degree of biodegradation in the planted soils (Supplementary Table 2).

### Biodegradation of polycyclic aromatic hydrocarbons

**Figure 4:**
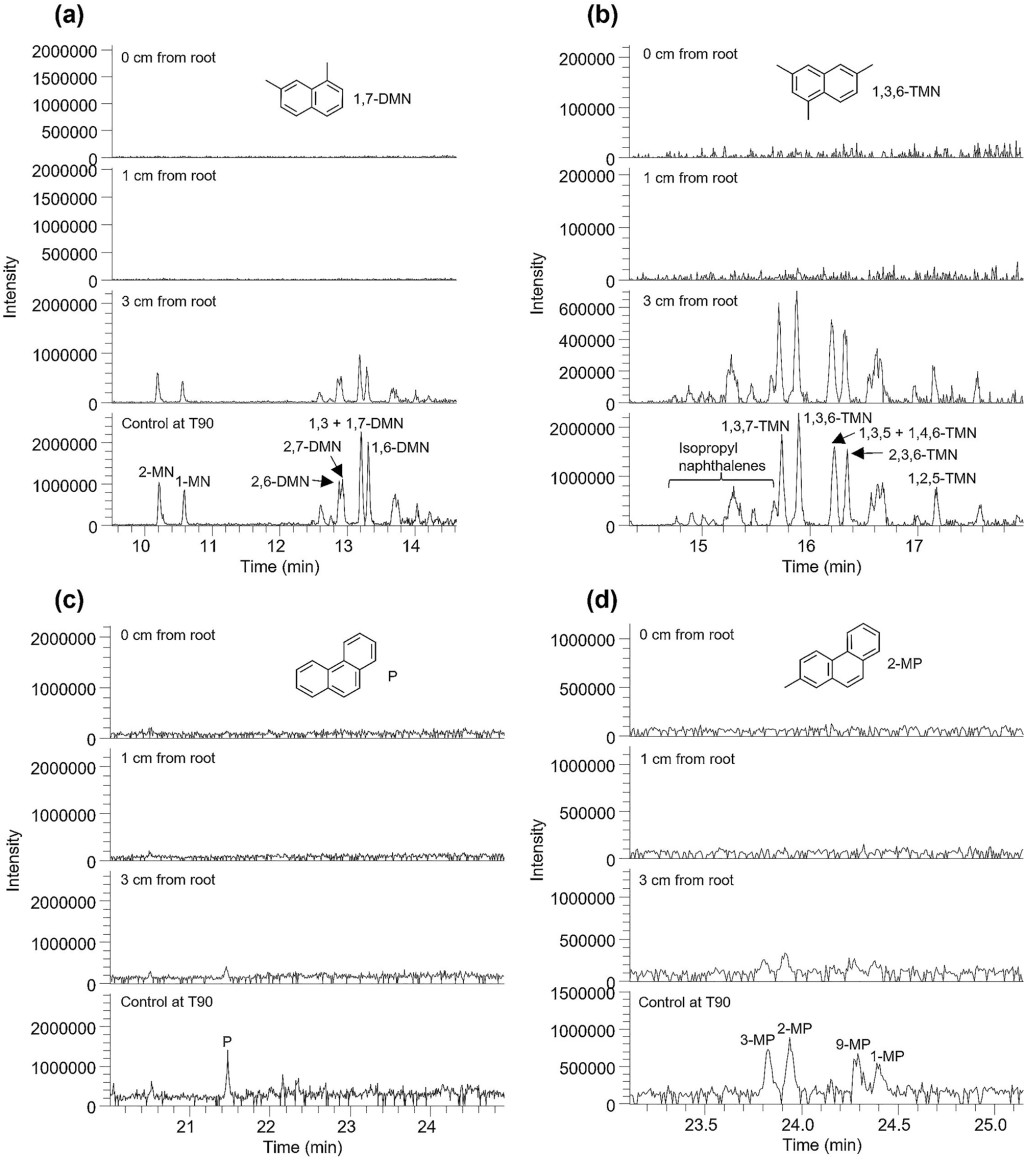
Biodegradation of polycyclic aromatic hydrocarbons as a function of distance from root surface. (**a**) Partial *m/z* 142 + 156 mass chromatograms of residual soils showing differential degradation of methylnaphthalenes (MN) and dimethylnaphthalene (DMN) respectively. (**b**) Partial *m/z* 170 mass chromatograms of residual soils showing differential degradation of trimethylnaphthalenes (TMN). (**c**) Partial *m/z* 178 mass chromatograms of residual soils showing differential degradation of phenanthrene (P). (**d**) Partial *m/z* 192 mass chromatograms of residual soils showing differential degradation of methylphenanthrene (MP). In (**a–d**), the chromatograms are normalized to sample weight and can be directly compared with reference to intensity values. Numbers denote positions of alkylation.

Gas chromatography–mass spectrometry analysis of residual polycyclic aromatic hydrocarbons showed near complete degradation of alkylnaphthalenes and alkylphenanthrenes such as dimethyl and trimethylnaphthalene, phenanthrene and methylphenanthrene (Fig. 4). It was observed that while mono-, di-, and tri-substituted alkylnaphthalenes were almost completely removed in soils at close proximity to the roots (0 cm and 1 cm from the root surfaces), it remained in bulk soil (3 cm from the root) (Fig. 4a, b). On the other hand, the dissipation of phenanthrene and monosubstituted alkylphenanthrenes were almost complete in planted soils even at 3 cm from the roots (Fig. 4c, d).

### Biodegradation parameters

**Figure 5:**
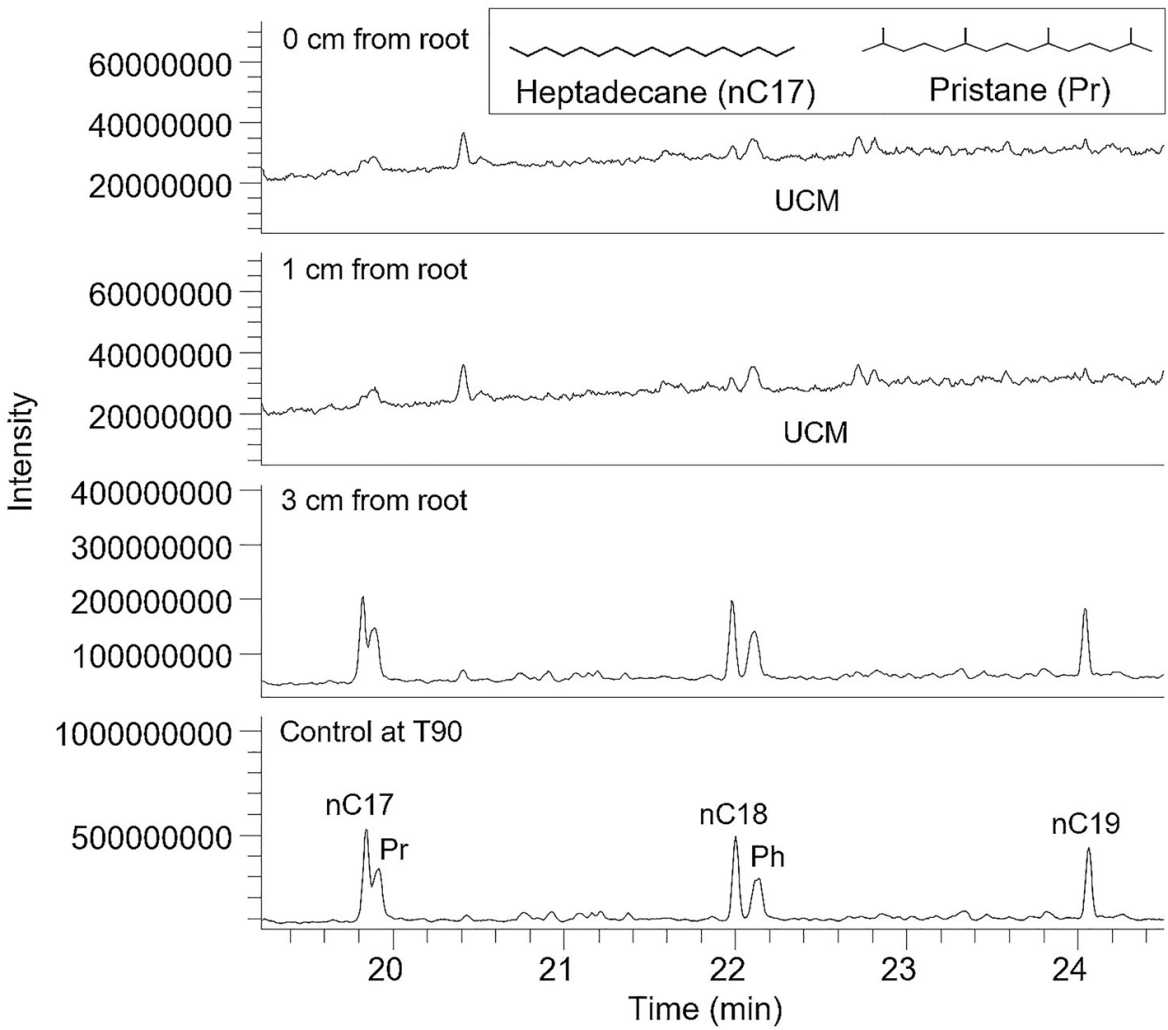
Partial total ion chromatograms showing preferential biodegradation of *n*C_17_ and *n*C_18_ relative to pristane (Pr) and phytane (Ph) with increasing biodegradation closer to root surfaces, a claim supported by huge unresolved complex mixture (UCM) seen in rhizosphere soils.

The greatest rate of biodegradation occurred in soils at close proximity to *M. sativa* roots as shown by the huge unresolved complex mixture (UCM) seen in soils at 0 cm and 1 cm from root surfaces (Fig. 5). This is further confirmed by the values of biodegradation ratios (Pr/*n*C_17_, Ph/*n*C_18_ and UCM/TPH). The values of these parameters at the end of the experiment for soils directly attached to *M. sativa* roots were 2.40, 2.55 and 2.83 for Pr/*n*C_17_, Ph/*n*C_18_ and UCM/TPH respectively (Supplementary Table 3). On the other hand, the lowest values of these ratios were seen in the unplanted control soil (control at T90) and the bulk soil (3 cm from root) (Supplementary Table 3).

### Relationship between root exudate concentration and hydrocarbon dissipation

**Figure 6:**
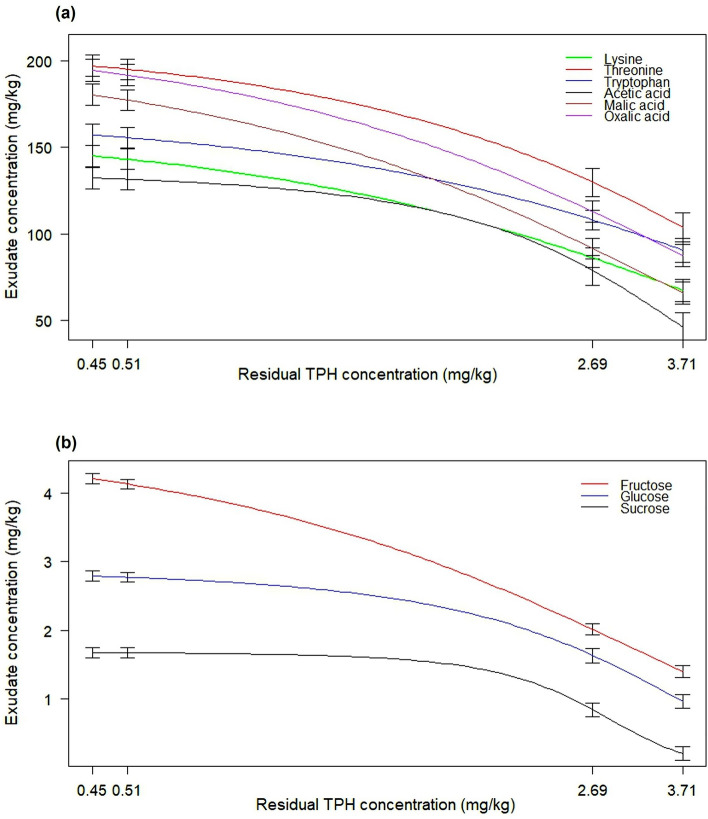
3-parameter logistic model showing the correlation between (**a**) amino/organic acids (mg/kg) and residual total petroleum hydrocarbons (mg/kg), and (**b**) soluble sugars (mg/kg) and residual total petroleum hydrocarbons (mg/kg). Plots show changes in residual TPH level in response to root exudate concentration. (Error bar stands for SE; n = 3).

The plot of root exudate concentration versus residual total petroleum hydrocarbon concentration reveals a significant negative correlation between root exudates and residual TPH, indicating that greater biodegradation (low residual TPH value) occurred at high concentration of root exudates (Fig. 6). The lowest rate of hydrocarbon dissipation was recorded at lowest concentration of root exudates, representing the unplanted control soil (control at T90).

### Root exudation of terpenoids in the rhizosphere

**Figure 7:**
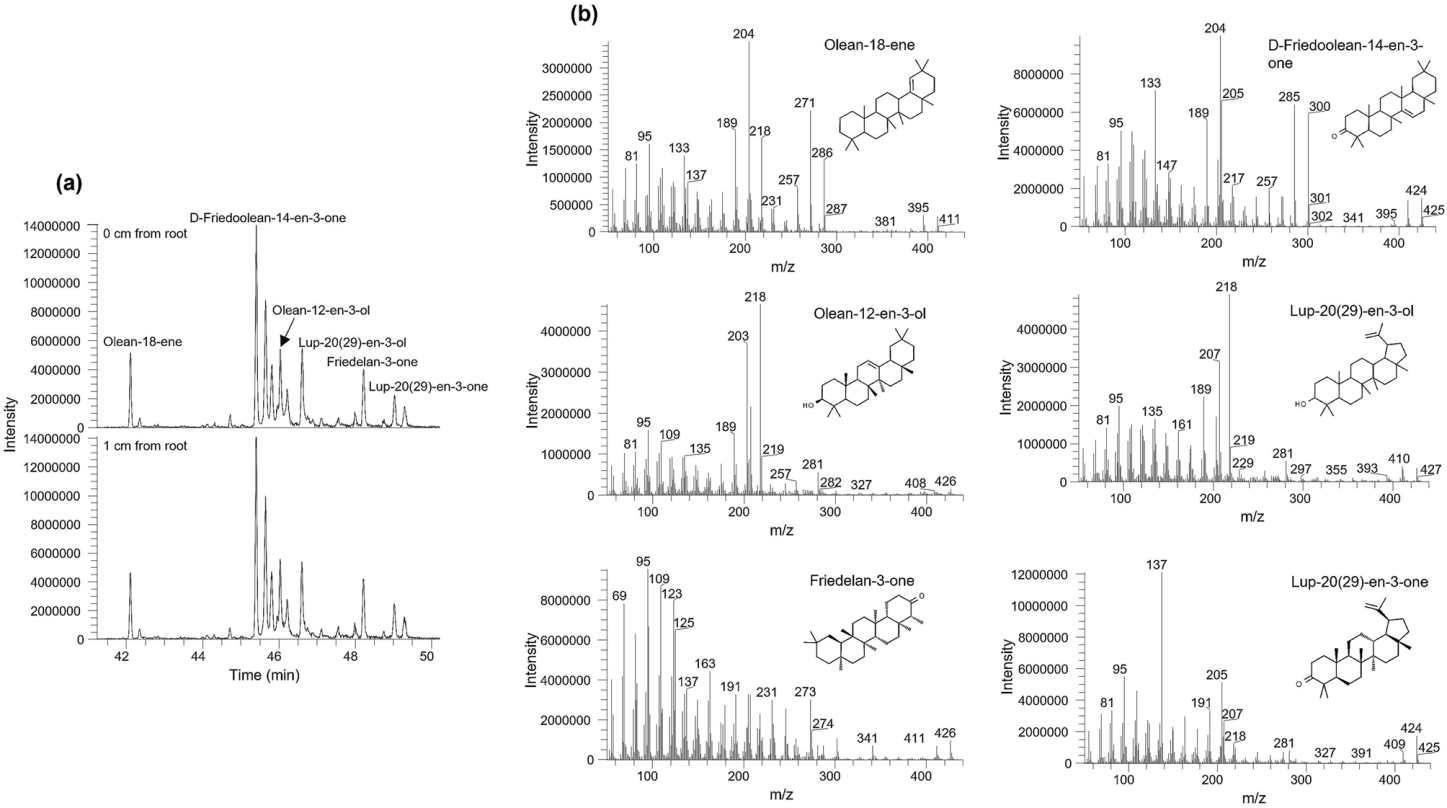
(**a**) Partial *m/z* 204 + 218 mass chromatograms and (**b**) mass spectra of selected terpenoids present in the rhizosphere of *M. sativa*.

Geochemical analysis revealed that with increasing distance from plant roots, there was a decreasing amount of terpenoids present in soil samples (Fig. 2a), indicating the contribution of *M. sativa* roots to soil terpenoids. Molecular analysis indicates the presence of, among others, the following terpenoids, otherwise known as isoprenoids: Olean-18-ene, D-Friedoolean-14-en-3-one, Olean-12-en-3-ol (also called ɑ-Amyrin), Lup-20(29)-en-3-ol (also called Lupeol), Friedelan-3-one, and Lup-20(29)-en-3-one (Fig. 7).

## Discussion

The remediation of environmental organic pollutants using plant-based techniques is an ecofriendly approach as it impacts minimally on soil matrix and associated microbiome. While plant can absorb and sequester organic contaminants through various mechanisms, the majority of hydrocarbon dissipation occur in the rhizosphere as a result of metabolic activities of soil-borne microbes. The results of this study revealed the role root exudates play in this process. In this study, *Medicago sativa* L. did not exhibit any visual sign of diesel fuel toxicity. This agrees with our previous study that revealed that although diesel fuel may lead to a lag in germination, it does not significantly affect *M. sativa* seed viability and growth rate^18^. On the contrary, low concentrations of hydrocarbons tend to exert a stimulatory influence on *M. sativa*, a concept known as hormesis^15,31,32^. The hormetic effect of hydrocarbons at low doses may also impact positively on root exudation.

Liquid chromatography-mass spectrometry analysis of the distribution of root exudates in the planted soils showed that the concentration of essential nutrients in soils correlated negatively with distance from *M. sativa* root surfaces (Fig. 1 and Supplementary Fig. 3). For example, while the concentrations of organic and amino acids were greatest in soils directly attached to the roots, their levels steadily declined with distance from root surfaces, reaching lowest concentrations in bulk soils (Table 1). In all cases, these values were significantly greater than those observed in the unplanted control soils. These findings agree with a previous study of ryegrass that showed that the concentration of oxalic acid and total soluble sugars decreased as the distance increased from plant roots^2^.

It can be inferred from the result of this work that the concentration gradient of root exudates in soil matrix is a function of three processes, namely exudation, diffusion (solubility) and biochemical transformation. It has been found that exudation, which refers to the quantity and quality of specific compound exuded by plant roots, is dependent on cultivar, plant species, growth stage and environmental factors, as well as the presence of microorganisms^33–35^. The results of this study revealed that the diffusion of exudates away from root surfaces is dependent mainly on compound solubility. For example, among compounds belonging to the same group (amino acids or organic acids), it was found that, except for malic acid, the slope of concentration gradient increased (equivalent to decreased diffusibility) with decreased solubility in water (Supplementary Table 1). The exceptionally low diffusibility of malic acid beyond 1 cm from root surfaces in comparison to other organic acids in this study may be attributed to its higher molecular mass and other unknown environmental factors. It was found in one previous study that the concentrations of root exudates in sterilized soil were higher than those of unsterilized soil, indicating that microbial consumption is an important determinant of exudate concentrations in soils^2^. Since rhizosphere microorganisms depend on root exudation for nutrition, the concentration of exudates at a given point within the soil matrix and away from root surfaces is generally the net from exudation, diffusion, and microbial consumption.

The analysis of residual total petroleum hydrocarbons revealed the positive effect of the presence of plant on the removal of petroleum hydrocarbons (Fig. 2). Previous studies have also alluded to the effectiveness of plants to remove organic and inorganic contaminants^11,36–38^. Plants orchestrate bioremediation through processes such as phytovolatilization, phytoextraction, phytostabilization and rhizodegradation^1,39,40^. The gradient of TPH away from root surfaces clearly showed that phytodegradation of pollutants is a function of contaminant proximity to root surfaces (Fig. 3). While very few researchers have examined the degradation of individual compounds such as phenanthrene in relation to root proximity^2,13,41^, to the best of our knowledge, this is the first study of the relationship between biodegradation of an entire petroleum distillate (diesel fuel) and proximity to root surfaces. It is also the first study that revealed the statistically significant correlation between exudate concentration and biodegradation level.

Molecular analysis of polycyclic aromatic hydrocarbons (alkylnaphthalenes, phenanthrene and alkylphenanthrenes) revealed concentration gradient away from root surfaces similar to that of total petroleum hydrocarbons. While these compounds were almost completely biodegraded in rhizosphere soils, their degradation declined with distance away from the rhizosphere and into the bulk soil, with least degradation observed in unplanted soil (Fig. 4). Similarly, Corgié, et al.^13^ observed that the degradation of phenanthrene decreased with distance away from *Lolium perenne* roots from 86% biodegradation at 0–3 mm from root surfaces to 36% biodegradation at 6–9 mm from roots. The enhanced rhizodegradation of these otherwise recalcitrant hydrocarbons at close proximity to root surfaces is a clear indication of the role of rhizosphere effect in contaminant dissipation. Rhizosphere effect can potentially be exploited for the clean-up of soils contaminated with highly resistant organic pollutants such as organochlorines and polychlorinated biphenyls.

The determined biodegradation ratios confirmed that the dissipation of hydrocarbons in this study resulted from biodegradation (Supplementary Table 3). The parameters Pr/*n*C_17_, Ph/*n*C_18_ and UCM/TPH followed the expected trend, namely, their values increased with increased degree of biodegradation. This is because of the preferential degradability of *n*-alkanes over the more refractory isoprenoid hydrocarbons^42^. The UCM is a recalcitrant product of biodegradation unresolvable by GC–MS. These ratios thus indicate that the greatest degree of biodegradation occurred in soils directly attached to *M. sativa* roots, while the least degree of biodegradation was in the unplanted control soil. This is further supported by the huge unresolved complex mixture (UCM) observed in rhizosphere soils in contrast to the bulk (3 cm from root) and the unplanted control soils (control at T90) (Fig. 5). As biodegradation proceeds, the abundances of major resolved compounds diminish, resulting in the more prominent chromatographic baseline hump termed unresolved complex mixture.

An important output of this study is the elucidation of the relationship between root exudate concentration and hydrocarbon dissipation. The results revealed negative correlations between the concentrations of root exudates and residual TPH levels (Fig. 6). Three mechanisms identified by previous researchers that may explain the reasons for the observed statistically significant relationship are the ability of root exudates to desorb hydrocarbons from soil making them bioavailable for microbial attack^43–45^; the ability of some root exudates to catalyse the oxidation of hydrocarbons into intermediate products^2^; and the ability of root exudates to serve as nutrients to soil microbes and consequently attract hydrocarbon-degrading microorganisms to the rhizosphere^46,47^. Root exudates have been found to alter microbial community composition and diversity in contaminated soils leading to a shift in metabolic activities^48,49^. Organic acids, amino acids and soluble sugars are some of the most important nutrient sources for hydrocarbon-degrading microbes^5,33^, and microorganisms have developed sensory systems that guide them to these compounds in order to satisfy their nutritional needs^50,51^. While not all the compounds that may influence biodegradation are derived from roots, the results of this study show that the presence of plant can significantly influence biodegradation of hydrocarbon pollutants through such root exudation as organic acids, amino acids, soluble sugars and terpenoids. Interestingly, in a recent study of the effects of root exudates on enzymatic activities of two oil degraders, *Micrococcus luteus* WN01 and *Bacillus cereus* W2301, Yang, et al.^52^ found that root exudates led to an increase in microbial population and dehydrogenase activity, and resulted in maximized removal of TPHs and PAHs. The relationship between root exudates and biodegradation observed in this study confirms the hypothesis that root exudates are ecological drivers of rhizodegradation.

The examination of terpenoids putatively exuded from *M. sativa* roots revealed structural similarity to polycyclic aromatic hydrocarbons (Fig. 7). Structural analogy improves hydrocarbon degradation by stimulating co-metabolism, which is the degradation of non-beneficial compounds along with beneficial ones^53–55^. For example, in a study of biomineralization of benzo[a]pyrene, Kanaly and Bartha^56^ found that co-metabolism was responsible for the initial steps of benzo[a]pyrene biodegradation. Similarly, phenols and phenylpropanoids were reported to support the degradation of polychlorinated biphenyls^54,57^. It seems that secondary metabolites especially terpenoids and flavonoids are significant drivers of biodegradation of recalcitrant organic compounds through co-metabolic processes^58,59^. Therefore, root exudates, including secondary metabolites, offer great prospects for biotechnological applications in the remediation of environmental organic pollutants, including recalcitrant polycyclic aromatic hydrocarbons.

In conclusion, as demonstrated in this experiment, the presence of plants can significantly influence biodegradation of diesel fuel hydrocarbons in contaminated soils. The concentration of root exudates within the soil matrix was shown to be a function of proximity to root surfaces. Similarly, biodegradation was found to correlate with distance from roots, ranging from ≥ 90% within the rhizosphere to < 50% in bulk soil and unplanted control soil. We believe that the results of this study will prove vital for future biotechnological exploitation of root exudates for increased biodegradation of organic pollutants, including recalcitrant ones.

### Supplementary Information

Supplementary Information.

## Data Availability

The raw datasets generated and analyzed during the current study have been deposited to figshare (https://doi.org/10.6084/m9.figshare.20400984.v1)^60^.
