# Supplementary material for: Elucidating the significant roles of root exudates in organic pollutant biotransformation within the rhizosphere
Source: Sci Rep. 2024 Jan 29;14:2359. doi: 10.1038/s41598-024-53027-x (PMC10824751; doi:10.1038/s41598-024-53027-x)
Supplement: Supplementary file 1 — Supplementary Information. [file 41598_2024_53027_MOESM1_ESM.pdf]

Elucidating the significant roles of root exudates in organic pollutant biotransformation within the rhizosphere

Michael O. Eze[1,2]* and Chinedu F. Amuji[3]

[1]Department of Chemistry, and Metabolomics and Environmental Toxicology Laboratory, Missouri University of Science and Technology, Rolla, MO 65409, USA.

[2]Centre for Research in Energy and Environment, Missouri University of Science and Technology, Rolla, MO 65409, USA.

[3]Department of Crop Science, University of Nigeria, Nsukka, Enugu State, Nigeria.

*Correspondence: meze@mst.edu

Supplementary Files

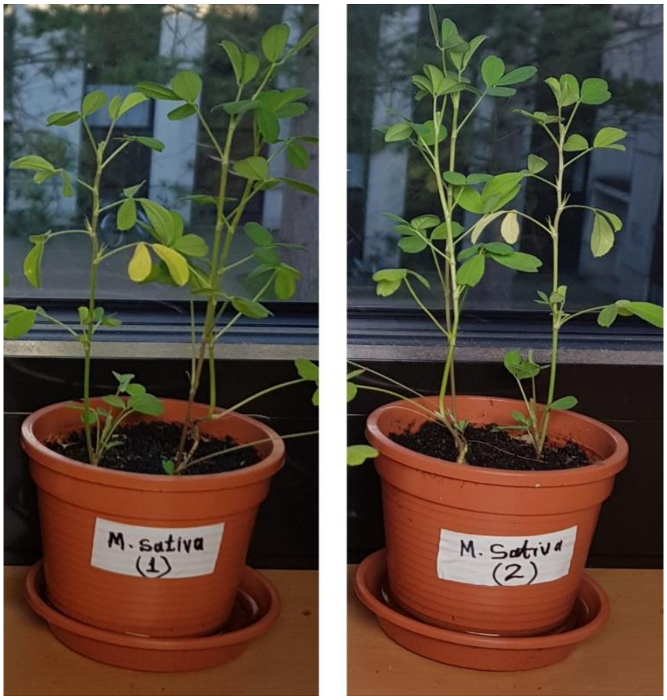

Supplementary Fig. 1. Pictures of *M. sativa* plants growing in hydrocarbon-contaminated soil.

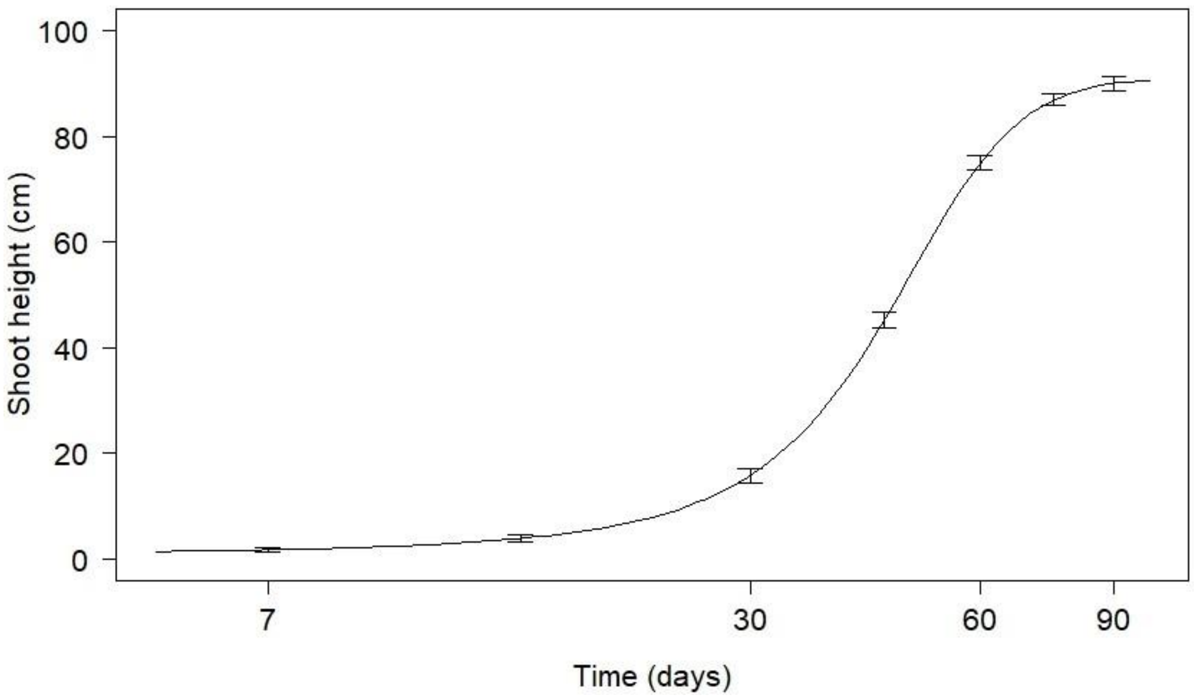

Supplementary Fig. 2. 3-parameter logistic model showing the growth of *M. sativa* (in terms of shoot height) in the diesel fuel-contaminated soil during the 90-day experimental period. (Error bar stands for SE; n = 3).

(a)

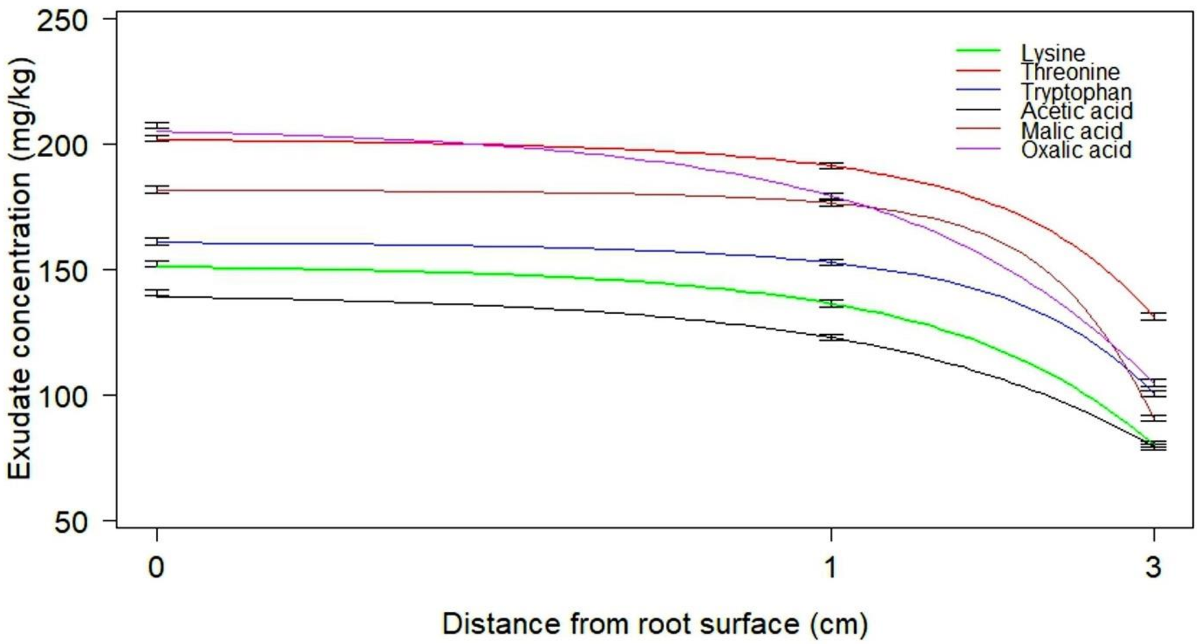

(b)

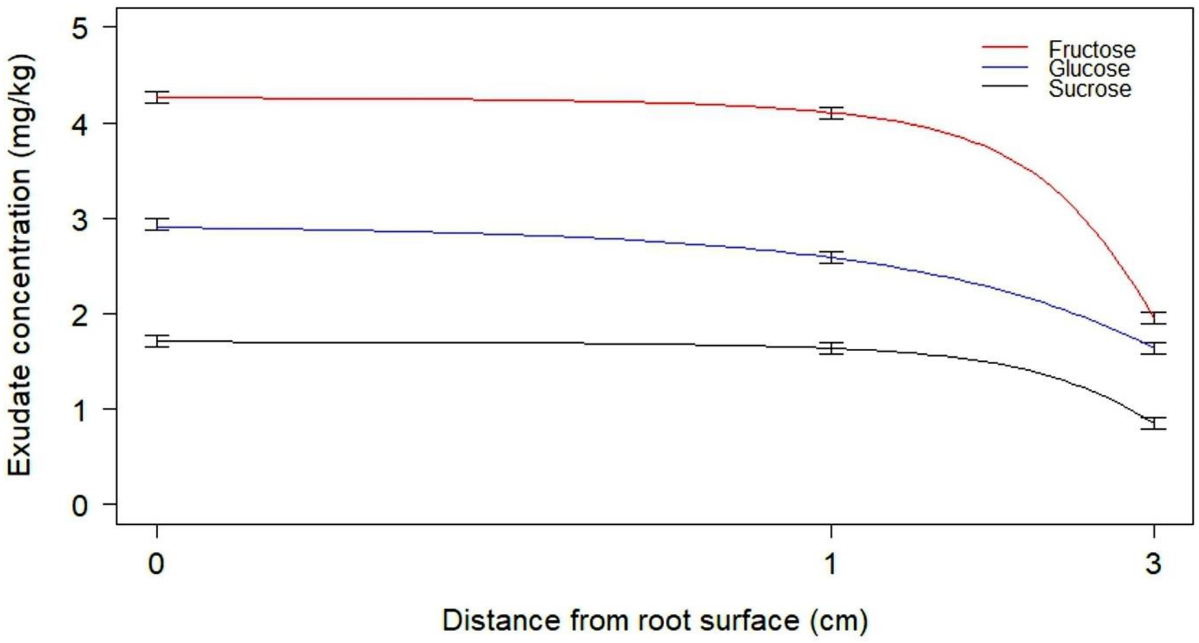

Supplementary Fig. 3. 3-parameter logistic model showing negative correlation between (a) amino/organic acids (mg/kg) and distance from root surfaces (cm), and (b) soluble sugars (mg/kg) and distance from root surfaces (cm). (Since root-dependent exudate concentration approaches zero at maximum distance, the lower asymptote, *c*, was fixed at 0 resulting in a 3-parameter logistic model).

Supplementary Table 1. The relationship between slope (*b* value) of the plot of exudate concentration versus distance and exudate solubility in water at 25 °C (g/L). Slope (*b*) was determined from the 3-parameter logistic model at 50% effect (*x* = 50%).

Compound	Molecular mass (g)	Solubility (g/L, 25 ° C)	Slope (*b*, $mgkg^{-1}cm^{-1}$)
Lysine	146.19	1000.0	0.76
Threonine	119.12	97.0	0.88
Tryptophan	204.22	11.4	0.98
Acetic acid	60.05	1000.0	0.49
Malic acid	134.09	592.0	1.64
Oxalic acid	90.03	220.0	0.61

Supplementary Table 2. Statistical parameters for one-way ANOVA showing significant differences between mean values of residual TPH at different distances from *M. sativa* root surfaces.

Linear hypothesis	Estimate	Std. Error	*t* value	Pr(>|t|)
(Intercept)	3.71333	0.08777	42.310	1.07e-10 ***
0 cm from root	-3.26000	0.12412	-26.265	4.74e-09 ***
1 cm from root	-3.20000	0.12412	-25.782	5.50e-09 ***
3 cm from root	-1.02000	0.12412	-8.218	3.60e-05 ***

Significant codes: 0 ‘***’ 0.001 ‘**’ 0.01 ‘*’ 0.05 ‘.’ 0.1 ‘ ’ 1.

Supplementary Table 3. Biodegradation parameters for the different treatments/distances from root surfaces.

Treatment/Distance	Pr/nC_{17}	Ph/nC_{18}	UCM/TPH
Soil at 0 cm from root	2.40	2.55	2.83
Soil at 1 cm from root	2.38	2.55	2.80
Soil at 3 cm from root	1.20	1.23	0.75
Control soil at T90	0.95	0.90	0.54
